# Cardiac manifestations of Fabry disease in G3Stg/*Gla*KO and *Gla*KO mouse models–Translation to Fabry disease patients

**DOI:** 10.1371/journal.pone.0304415

**Published:** 2024-05-31

**Authors:** Abirami Kugadas, Pietro Artoni, Wanida Ruangsiriluk, Meng Zhao, Natalia Boukharov, Rizwana Islam, Dmitri Volfson, Katayoun Derakhchan

**Affiliations:** 1 Rare Diseases Drug Discovery Unit, Takeda Development Center Americas Inc., Cambridge, Massachusetts, United States of America; 2 Oncology and Immunology Unit, WuXi AppTec, Natick, Massachusetts, United States of America; 3 Statistical and Quantitative Sciences, Takeda Development Center Americas Inc., Cambridge, Massachusetts, United States of America; 4 Crosswalk Therapeutics, Cambridge, Massachusetts, United States of America; 5 Pioneering Medicines at Flagship Pioneering, Cambridge, Massachusetts, United States of America; Weizmann Institute of Science, ISRAEL

## Abstract

Fabry disease (FD) is an X-linked disorder of glycosphingolipid metabolism caused by mutations in the GLA gene encoding alpha-galactosidase A (α-Gal). Loss of α-Gal activity leads to progressive lysosomal accumulation of α-Gal substrate, predominately globotriaosylceramide (Gb3) and its deacylated derivative globotriaosylsphingosine (lyso‐Gb3). FD manifestations include early onset neuropathic pain, gastrointestinal symptoms, and later onset life-threatening renal, cardiovascular and cerebrovascular disorders. Current treatments can preserve kidney function but are not very effective in preventing progression of cardiovascular pathology which remains the most common cause of premature death in FD patients. There is a significant need for a translational model that could be used for testing cardiac efficacy of new drugs. Two mouse models of FD have been developed. The α-Gal A-knockout (*Gla*KO) model is characterized by progressive tissue accumulation of Gb3 and lyso-Gb3 but does not develop any Fabry pathology besides mild peripheral neuropathy. Reports of minor cardiac function abnormalities in *Gla*KO model are inconsistent between different studies. Recently, G3Stg/*Gla*KO was generated by crossbreeding *Gla*KO with transgenic mice expressing human Gb3 synthase. G3Stg/*Gla*KO demonstrate higher tissue substrate accumulation and develop cellular and tissue pathologies. Functional renal pathology analogous to that found in early stages of FD has also been described in this model. The objective of this study is to characterize cardiac phenotype in *Gla*KO and G3Stg/*Gla*KO mice using echocardiography. Longitudinal assessments of cardiac wall thickness, mass and function were performed in *Gla*KO and wild-type (WT) littermate controls from 5–13 months of age. G3Stg/*Gla*KO and WT mice were assessed between 27–28 weeks of age due to their shortened lifespan. Several cardiomyopathy characteristics of early Fabry pathology were found in *Gla*KO mice, including mild cardiomegaly [up-to-25% increase in left ventricular (LV mass)] with no significant LV wall thickening. The LV internal diameter was significantly wider (up-to-24% increase at 9-months), when compared to the age-matched WT. In addition, there were significant increases in the end-systolic, end-diastolic volumes and stroke volume, suggesting volume overload. Significant reduction in Global longitudinal strain (GLS) measuring local myofiber contractility of the LV was also detected at 13-months. Similar GLS reduction was also reported in FD patients. Parameters such as ejection fraction, fractional shortening and cardiac output were either only slightly affected or were not different from controls. On the other hand, some of the cardiac findings in G3Stg/*Gla*KO mice were inconsistent with Fabry cardiomyopathy seen in FD patients. This could be potentially an artifact of the Gb3 synthase overexpression under a strong ubiquitous promoter. In conclusion, *Gla*KO mouse model presents mild cardiomegaly, mild cardiac dysfunction, but significant cardiac volume overload and functional changes in GLS that can be used as translational biomarkers to determine cardiac efficacy of novel treatment modalities. The level of tissue Gb3 accumulation in G3Stg/*Gla*KO mouse more closely recapitulates the level of substrate accumulation in FD patients and may provide better translatability of the efficacy of new therapeutics in clearing pathological substrates from cardiac tissues. But interpretation of the effect of treatment on cardiac structure and function in this model should be approached with caution.

## Introduction

Fabry disease (FD) is a lysosomal storage disorder caused by deficiency of the enzyme α-Gal due to mutations in the GLA gene located on the X chromosome. Deficiency of α-Gal leads to accumulation of glycosphingolipids, in various cell types throughout the body [1], mainly affecting the kidneys, heart, and nervous system. The disease can be divided into a severe, classical phenotype, diagnosed in early childhood, and most often seen in males with residual enzyme activity of <1%, and a milder, later onset nonclassical phenotype. Classical FD manifestations include neuropathic pain, gastrointestinal dysfunction, progressive renal failure, hypertrophic cardiomyopathy, cardiac rhythm disturbances, and stroke [2]. Nonclassical FD is characterized by later onset, variable disease course with manifestations that may be limited to a subset of those affecting classical patients or even to a single pathology. For example, IVS4+919G>A mutation in Taiwanese FD patients and p. N215S in UK mostly cause late onset cardiac pathology [3]. With early treatment initiation, renal function can be preserved with enzyme replacement therapy (ERT) and modulators of renin-angiotensin-aldosterone system [4]. But even with stabilization of kidney function, patients still experience the same cardiac events as have been described in untreated patients [5]. New treatments for FD are currently being developed but nonclinical testing of the potential of these treatments to address unmet needs such as progression of cardiac pathology is impeded due to the lack of relevant nonclinical translational biomarkers [6, 7]. Mouse *Gla* knockout Fabry model (*Gla*KO) is widely used for testing efficacy of novel therapeutic modalities. This model is characterized by progressive substrate accumulation similar to FD patients but does not develop the same functional pathologies. Minor cardiovascular alterations were found in this model by several investigators [7–12]. Recently, a symptomatic Fabry mouse model expressing Gb3 synthase transgene on the *Gla* knockout background (G3Stg/*Gla*KO) has been developed. Tissue substrate accumulation in this model is significantly higher (10 times) than that in the *Gla*KO mice and renal symptomology characteristic of early stages of FD has been reported [13, 14]. There are no reports on any pathological changes in cardiac structure or function.

In the present study we aimed to assess these two FD mouse models in order to identify translatable functional endpoints that can be used to predict potential cardiac efficacy of novel treatments in FD patients.

## Methods

### Animals

All studies in animals were performed in accordance with the Guide for the Care and Use of Laboratory Animals and were approved by the Institution Animal Care and Use Committee (IACUC). The nbio232 (TgG3S) mouse line was supplied by JCRB Laboratory Animal Resource Bank of the National Institutes of Biomedical Innovation, Health and Nutrition, a company organized under the laws of Japan having a place of business at Saito-Asagi 7-6-8, Ibaraki, Osaka 567–0085, Japan (NIBIOHN). We have generated the G3Stg/*Gla*KO (G3StgGLAko) mouse model by crossing them to *Gla*KO mice which were acquired from the Jackson Laboratory (RRID:IMSR_JAX:003535 B6;129-Glatm1Kul/J also known as α-Gal A KO) [13]. These mice carried a knockout mutation in the galactosidase alpha (*Gla*) gene located in the X-chromosome, and were hemizygote and abbreviated as HEMI:WT. The G3Stg/*Gla*KO was *Gla*KO hemizygote and a carrier (CAR) for the transgene, therefore abbreviated as HEMI:CAR. Finally, there was one control WT group abbreviated as WT:WT. These two groups of mice had the same C57BL6/129sv genetic background, and their colonies were maintained at Taconic Biosciences. A pilot Study was carried out at the Life Science Research Institute (LSRI) animal facility/AGADA Biosciences, Halifax, NS, Canada. Male *Gla*KO (HEMI:WT), G3Stg/*Gla*KO (HEMI:CAR), and C57BL6/129sv (WT:WT) matched strain background WT mice at 12–15 weeks of age were shipped to LSRI and acclimated for at least 7 days before the start of the study. Immediately after acclimation, the mice were weighed. From there, body scores were taken daily, and body weights were collected weekly. If a mouse had more than a 20% reduction in body weight or a body score of 16 or higher, it was euthanized to relieve unnecessary pain or distress, based on the veterinarian’s recommendation. Supplementary to the aforementioned study, cardiac assessments were investigated by echocardiography in male G3Stg/*Gla*KO (HEMI:CAR) mice and littermate controls (WT:WT) between 22 to 28 weeks of age in a subsequent Study #1. In Study #2, cardiac assessments were performed by serial echocardiography (once per month) in male *Gla*KO (HEMI:WT) mice and littermate controls (WT:WT) from 5 to 13 months. Both Studies #1 and #2 were executed in house after animals were shipped from Taconic Biosciences and acclimated on site.

Animals were sacrificed by carbon dioxide (CO_2_) inhalation followed by a secondary method of euthanasia, such as decapitation, bilateral pneumothorax, or cervical dislocation.

### Body scores

A total body score representing the overall phenotypic and symptomatic assessment for each animal was obtained by scoring each mouse on six phenotypic parameters. Mice were phenotypically assessed for body thinness (also known as body conditioning), hunched appearance or kyphosis, tremors, hindlimb clasping, disturbances in gait, and overall movements such as circling, seizures, difficulty ambulating, or spinning in circles when lifted by the tail. These features were monitored daily, starting when mice were 16 weeks of age until takedown, when the mice were 30 weeks of age. A numerical value of 1 = normal, 2 = mild/intermediate (animals presenting some degree of the phenotype), and 3 = severe deficit was given for each assessment (Table 1).

**Table 1:** Body condition criteria.

Component/Score	1	2	3
**Hindlimb Clasping**	When animal lifted near the base of the tail and suspended in mid-air for 10 seconds, hindlimbs consistently splayed outward and away from the abdomen.	When animal lifted near the base of the tail and suspended in mid-air for 10 seconds, one or both hindlimbs retracted towards the abdomen for less than 50% of the time suspended.	When animal lifted near the base of the tail and suspended in mid-air for 10 seconds, one or both hindlimbs entirely retracted and touching the abdomen for more than 50% of the time suspended.
**Body Condition/Thin Appearance**	Vertebrae and dorsal pelvis not prominent, palpable with slight pressure.	Segmentation of vertebral column evident. Dorsal pelvic bones readily palpable.	Skeletal structure extremely prominent, little or no flesh cover. Vertebrae distinctly segmented.
**Gait Disturbances**	When mouse observed from behind as it walked, mouse moved normally with body weight supported on all limbs and abdomen not touching the ground. Hindlimbs participated evenly during walking.	Tremor and limping observed as mouse walked, lowered pelvis or feet pointed away from the body during movement.	Mouse had difficulty ambulating or moving normally, it dragged its abdomen on the surface.
**Tremors**	No observable tremors when mouse observed for 1–2 minutes.	Animal had less than two tremors when observed for 1–2 minutes.	Animal had more than two tremors when observed for 1–2 minutes or was shaking uncontrollably.
**Hunched Appearance (Kyphosis)**	Animal ambulated normally and straightened its spine when walking.	Animal had mild hunched posture but able to straighten its spine during walking, or unable to straighten its spine but mild hunched posture.	Mouse unable to ambulate normally, maintained pronounced hunched appearance during walking or siting.
**Movement observations**	Inside the cage, animal interacted with cage mates, was eating, drinking, and nesting.	Inside cage, animal had difficulty ambulating, slow/sluggish but active, slight degree of possible neurological issues.	Severe symptoms of neurological issues (i.e.: spinning or circling inside the cage, severe heat tilt, refusal to eat/drink/unable to move).

A score for each component was recorded and a composite score was calculated as a sum of individual component scores weekly. Normal mice were given a value of 1 for each of the six components assessed, setting a baseline score at 6, while mice exhibiting severe abnormalities for all six components could score as high as 18. However, to eliminate unnecessary pain or distress, any animal scoring 16 or greater was euthanized.

### Echocardiography

Parasternal long axis and short axis images were acquired using a Vevo3100 machine (FujiFilms, Visualsonics, Ontario, Canada) equipped with a MX550S probe. On the day of the procedure, animals were anesthetized with 5% isoflurane via induction chamber and echocardiography was performed on anesthetized mice maintained with ~1.5–2% isoflurane with a cone mask. Fur on the ventral thoracic region was removed using a clipper and depilatory agent such as Nair. All the depilatory agent that was applied was removed completely. Mice were then placed on dorsal recumbency and placed on the mouse imaging platform which has a heating plate that is prewarmed. Non-irritating tape was used to securely position the animal. Electrocardiogram (ECG) gel was applied on the animal paws, each paw was then connected to an ECG electrode. A little amount of lubricant was applied on the rectal probe and the probe was positioned in the rectum. The ECG, heart rate (HR), respiration frequency and rectal temperature were monitored continuously. Ultrasound gel was applied on the ventral thoracic region. When the animal was secured and stable, the transducer was positioned, and appropriate settings were selected for data acquisition. The probe can be manually held or connected to the stand to allow hands-free acquisition of data. First, the B-mode setting was used to collect long axis readouts. This was followed by EKV and M-mode short axis image acquisitions. Different parameters require slightly different positioning of the platform and transducer, and different settings such as B-mode, M-mode, doppler, and speckle tracking, but the animal remained in the same secured position on the platform. Parameters such as heart rate and volume (ejection fraction, stroke volume, end-diastolic volume, and end-systolic volume) were extracted from long axis B-mode. Fractional shortening, cardiac output, and LV wall thickness were measured/calculated from short axis M-mode. The entire procedure typically would take between 10–20 minutes. Once data collection was completed, the animal was removed from the platform and the ultrasound gel removed from the skin by gently wiping it away. The animals were then placed back into their home cage and observed until they recover from anesthesia and are ambulatory. Both long and short axis images were analyzed using Vevo Lab (5.5.0). An average of three heartbeats that occurred in between the respirations were analyzed to exclude any artifacts caused by the respiratory movements. A total of 26 parameter output was achieved using both long and short axis images. Global longitudinal strain (GLS) was measured using the same Vevo Lab (5.5.0) Strain module on the LV long axis images, and data analysis on the strain curves was performed using Matlab and R. For the analyses, the evaluator was blinded to the genotype of the mice, and the sequence of analysis was randomized. In addition, random images from all groups were quality-controlled and evaluated blindly by another evaluator and found no difference between the evaluators.

### Statistical analysis

Statistical analysis (stats) of the data was conducted using the R programming language and GraphPad Prism software. Our approach incorporated longitudinal mixed modeling in case of longitudinal data and repeated measures. In the case of longitudinal statistical modeling, p values were corrected using the Tukey method to control the Family wise error rate. In the case of simple t tests across multiple types of measurements (not longitudinal), the p values were corrected using the conservative Bonferroni correction. The nominal level for defining statistical significance is p ≤ 0.05. Normality was tested within each group using both the Shapiro-Wilk normality test and a visual inspection of each histogram.

Statistical analysis on data represented in Supplementary S2 and S3 Figs in S1 Appendix, and in Supplementary S1-S3 Tables in S1 Appendix: Comparisons between each phenotype were performed using two-sample student’s t-tests for equal variances. This analysis was analogous to an ANOVA with a Dunnett’s post-hoc test to account for all groups compared to WT control (WT:WT). The p values were adjusted because of multiple comparisons using Bonferroni correction. Analysis of body weight and body scores were performed at each time point and over time. Comparisons at each time point utilized a student’s t-test. Analysis of body weight and body scores over time used longitudinal linear regression models which accounted for repeated measurements within each mouse. Two model terms were analyzed: group term and time term. Group term determined if there were significant differences between WT:WT compared to disease groups throughout the study period over all timepoints combined, while time term determined if there were significant differences from 15 to 30 weeks of age for the combined group effect. All stats of longitudinal models were based on observed values, rather than adjusted values normalized to the baseline that were displayed in the figures.

Statistical analysis on data represented in Figs 2, 4 and 5, and in Supplementary S4 and S7 Tables in S1 Appendix: P values have been calculated using 2-sample Student t-test (Welch’s t-test for unequal variances), and then corrected for multiple comparisons (Bonferroni).

Statistical analysis on data represented in Fig 3, and in Supplementary S5 and S6 Tables in S1 Appendix: We used longitudinal mixed modeling to account for repeated measurements over time. Data have been modeled using linear mixed modeling after log transformation, then deriving contrasts. log(feature) ~ genotype * month + (1|mouse). Genotype and month are both factors and have been modeled as a fixed effect, while the mouse ID has been modeled a random intercept. Contrasts from the statistical model have been evaluated with emmeans package in R, and p values were adjusted using the Tukey method to control the family wise error rate (FWER).

## Results

### Study design

#### Pilot study with both Fabry mouse models

Three groups of age-matched animals were enrolled in this study: Fifteen *Gla*KO (HEMI:WT), fourteen G3Stg/*Gla*KO (HEMI:CAR), and fifteen C57BL6/129sv matched strain background WT mice (Table 2).

**Table 2:** Pilot study design.

Group	Code	Genotype	Sex	Number	Age at baseline (weeks)	Age at study termination (weeks)
1	WT:WT	C57BL6/129sv	Male	15	15–16	30–31
2	HEMI:WT	*Gla*KO	Male	15	15–16	30–31
3	HEMI:CAR	G3Stg/*Gla*KO	Male	14	15–16	30–31

The study began when mice were 15–16 weeks old and continued until all mice reached 30–31 weeks of age.

#### Cardiac assessments in G3Stg/*Gla*KO Fabry mouse model (Study #1)

Twenty five HEMI:CAR mice and twenty five of their WT:WT littermate controls were enrolled in this study (Table 3). Echocardiography assessments were performed once when they reached 28 weeks of age. In three mice in the HEMI:CAR group, echocardiography was performed before euthanasia, at 22–25 weeks of age, due to severe dermatitis and body weight loss.

**Table 3:** Study design–Cardiac assessments in G3Stg/*Gla*KO mice.

Group	Code	Genotype	Sex	Number	Age at Terminal Echocardiography (weeks)
1	WT:WT	C57BL6/129sv	Male	25	28
2	HEMI:CAR	G3Stg/*Gla*KO	Male	25	22–28

#### Cardiac assessments in *Gla*KO Fabry mouse model (Study #2)

Twenty HEMI:WT and twenty one littermate WT:WT control mice were enrolled in this study. After the mice were acclimated, serial echocardiography assessments were performed once monthly in 5–13 months old *Gla*KO and age-matched littermate mice for a total duration of 9 months (Table 4 and S1 Fig).

**Table 4:** Study design–Cardiac assessments in *Gla*KO mice.

Group	Code	Genotype	Sex	Number	Age at Start Echocardiography (months)	Age at Terminal Echocardiography (months)
1	WT:WT	C57BL6/129sv	Male	21	5	13
2	HEMI:WT	*Gla*KO	Male	20	5	13

### G3Stg/*Gla*KO and *Gla*KO Fabry mice phenotype

Natural history data were collected in the pilot study (Table 1). Although the average body weight of HEMI:WT (*Gla*KO) mice was significantly higher in the initial weeks of the study (S1 Table in S1 Appendix), these mice had a slower longitudinal percent increase in body weight compared to the WT counterpart (S2 Fig in S1 Appendix). Unlike WT:WT and HEMI:WT mice that gain 10%-20% weight by the end of the study, HEMI:CAR exhibited progressive weight loss: the average body weight of HEMI:CAR mice was not significantly different compared to WT:WT from 15 to 21 weeks of age (S1 Table in S1 Appendix). However, by 22 weeks of age, the average body weight of HEMI:CAR mice was significantly lower (mean ± SD: 27.80 ± 4.69 g, p = 0.0289) to that of WT:WT (32.01 ± 3.83 g) mice, which persisted until the end of the study. The percent change in the body weight of HEMI:CAR mice over time showed a steady decline in body weight (S2 Fig in S1 Appendix).

We performed longitudinal analyses with repeated measurements, keeping in consideration a statistical model with a time factor and a group factor. When comparing the body weights of the FD mice and the WT:WT throughout the study period (group term), there was a significant difference (p = 0.026; ANOVA type III, S2 Table in S1 Appendix) in the slope, suggesting that there is a group factor that likely explains the variance. In addition, the significance on the time term (p<0.0001; S2 Table in S1 Appendix) suggests that considering different slopes in weight development are necessary to explain the variance in such longitudinal data.

Consistent with the body weight loss, body scores of HEMI:CAR mice also worsened rapidly with age. By 16 weeks of age, HEMI:CAR mice already exhibited significantly higher body score values when compared to their WT counterparts (6.64 ± 0.93 versus 6.00 ± 0.00, p = 0.024). A steady increase in the average body scores of HEMI:CAR was observed in subsequent weeks (S3 Fig S1 Appendix).

Due to disease progression and reaching the body score of 16 or higher one HEMI:CAR mouse had to be euthanized at 23 weeks of age, followed by five more at ages between 24 and 28 weeks. In contrast to the rapid progression of phenotypic worsening observed in HEMI:CAR mice, HEMI:WT mice maintained normal appearance and function through week 22 with minor abnormalities observed thereafter. Nevertheless, although small, increases in body scores in HEMI:WT mice were statistically significant when compared to the WT controls starting at 23 weeks of age (6.80 ± 0.56 versus 6.07 ± 0.26, p = 0.0002) (S3 Fig and S3 Table in S1 Appendix).

### Symptomatic Fabry G3Stg/*Gla*KO mice exhibit hypertrophic hypercontractile cardiomyopathy

**Fig 1:**
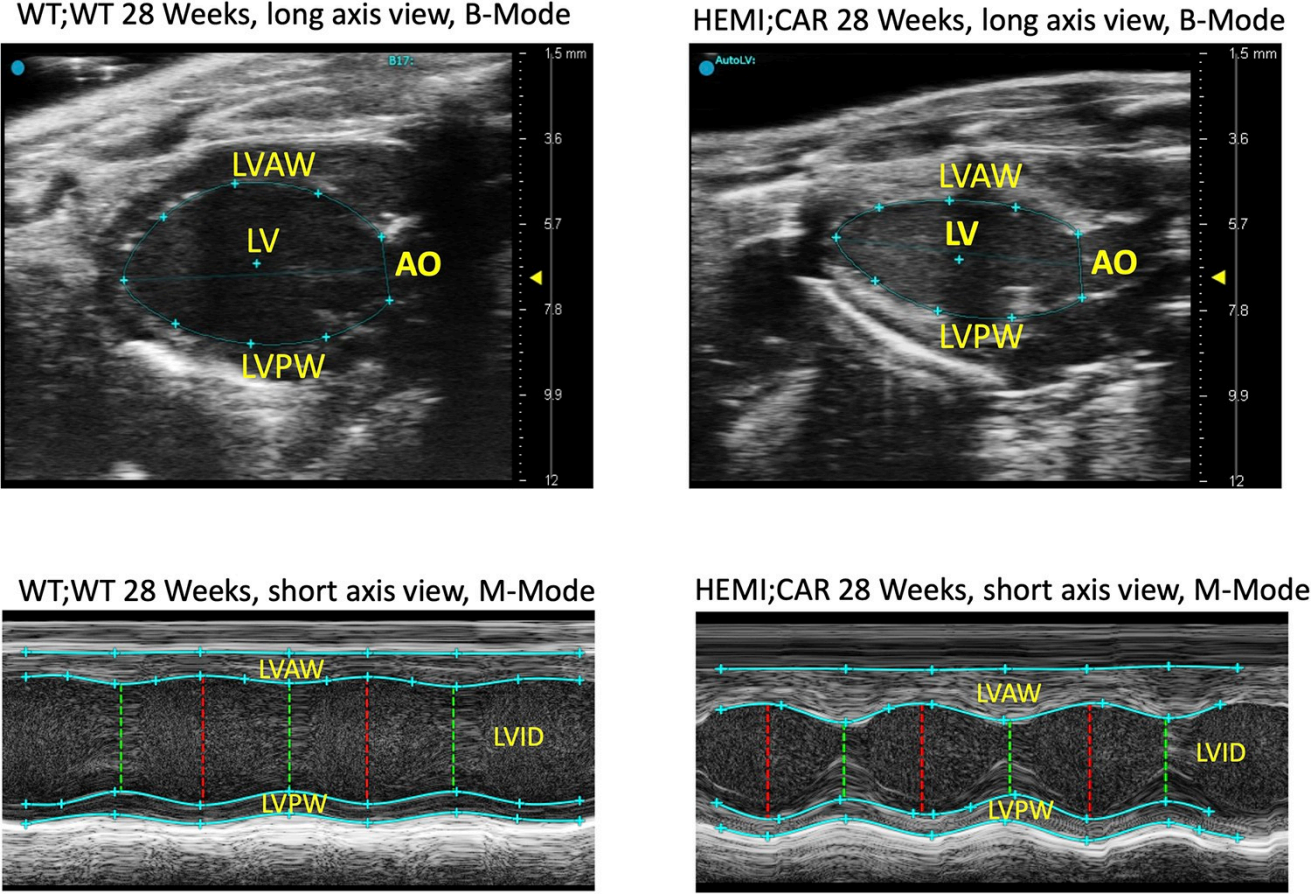
Representative echocardiography images: B-Mode and M-Mode in a G3Stg/*Gla*KO mouse and an age-matched WT:WT mouse. Abbreviations: AO = Thoracic aorta, LV = Left ventricle, LVAW = Left ventricular anterior wall, LVID = Left ventricular internal diameter, LVPW = Left ventricular posterior wall.

Echocardiography was performed on HEMI:CAR mice at 27–28 weeks and WT:WT mice at 28 weeks (Table 2, Study #1). Three HEMI:CAR mice were sacrificed earlier (age 22–25 weeks) due to disease progression with severe dermatitis and body weight loss, and echocardiography was performed before euthanasia and included in the comparison. HEMI:CAR mice were found to have hypertrophic hypercontractile cardiomyopathy (Fig 1).

**Fig 2:**
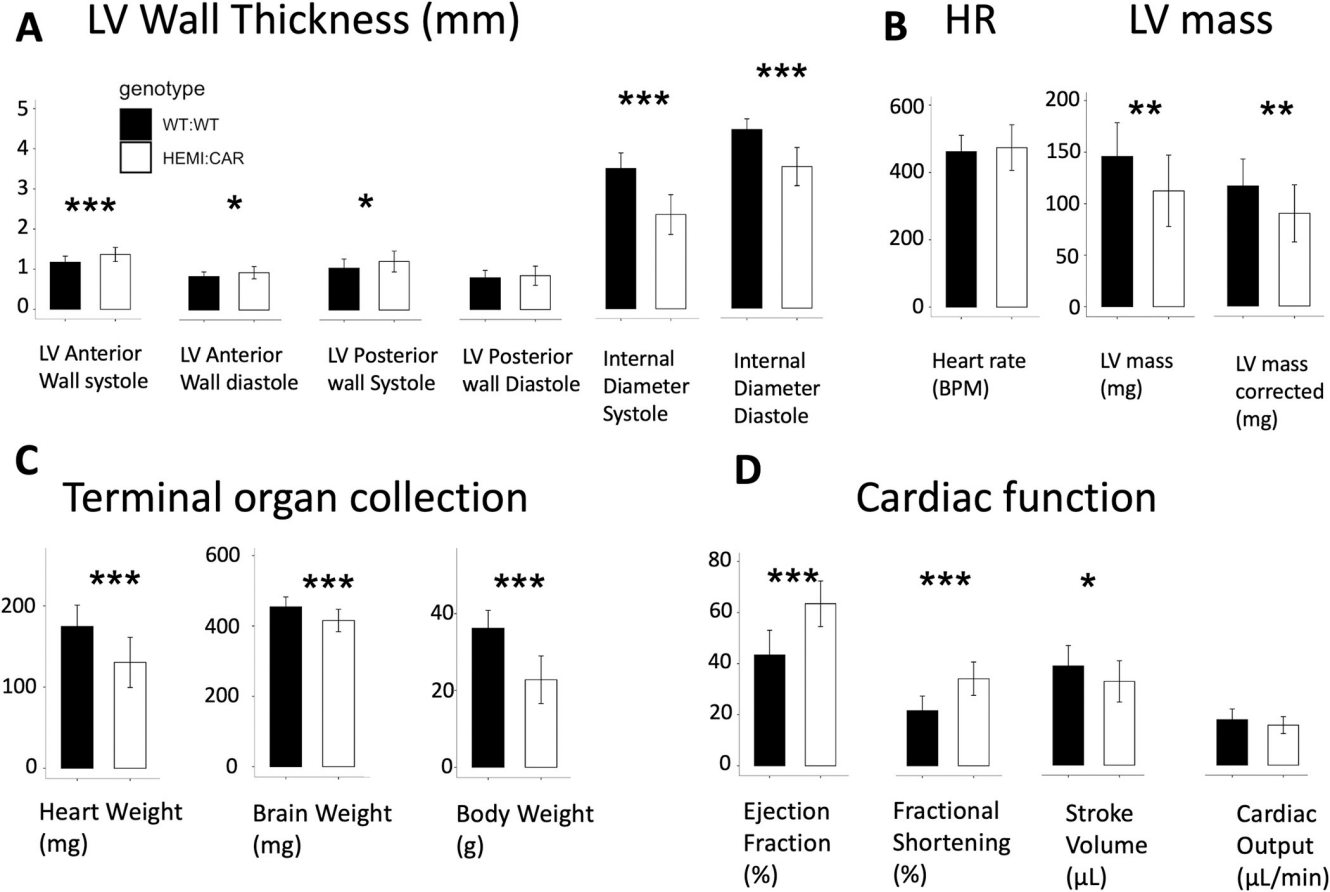
Characterization of cardiac function, left ventricular (LV) wall thickness, heart rate (HR), LV mass and organ weight in HEMI: CAR and age-matched WT: WT. For values and stats, see S4 Table in S1 Appendix. * p ≤0.05, ** p ≤0.01, *** p ≤0.001. The p values are Bonferroni corrected. Number of animals per group = 25.

The LV anterior and posterior wall at systole and diastole in HEMI:CAR mice was thicker by up to 16%, with significant reductions in the LV internal diameter by up to 33% (2.35 ± 0.50 mm, at systole), when compared to the age-matched WT:WT mice (3.50 ± 0.38 mm, at systole, p = 7e-12) (Fig 2A).

Surprisingly, the LV mass and corrected LV mass of the HEMI:CAR mice were significantly lower in WT:WT mice by 23% (LV mass: 145.8 ± 32.8 mg vs 112.4 ± 34.7 mg, p = 0.00104) (Fig 2B), and there was a significant decrease (p = 1.6e-6) in their heart weights by 25% when compared to WT:WT mice (Fig 2C). There was also significant increases by up to 40% in the ejection fraction (HEMI:CAR 58.8 ± 10.7% vs WT:WT 42.0 ± 11.4%, p = 9e-10), and up to 58% increase in fractional shortening (HEMI:CAR 34.1 ± 6.5% vs WT:WT 21.5 ± 5.7%, p = 4e-9) (Fig 2D). In HEMI:CAR mice, HR was slightly increased (Fig 2B), stroke volume significantly decreased and there was no change in cardiac output (Fig 2D). This increase in force of contraction (ejection fraction and fractional shortening) is probably due to the increase in the wall thickness and decrease in heart weight, and probably not due to changes in myocardial contractility. HEMI:CAR mice also presented with a 37% lower average terminal body weight (p = 4e-11) and 8% reduction in the average brain weight (p = 4e-5), when compared to WT:WT controls (Fig 2C).

We have also thoroughly studied the cardiac phenotype of these symptomatic Fabry mouse model in the pilot study. Echocardiography analysis was performed for HEMI:CAR (N = 14) and aged-matched WT:WT (N = 15) groups at 6 timepoints, every three weeks, corresponding to the mice at 15, 18, 21, 24, 27, and 30 weeks of age (data not shown). At younger age of 15–21 weeks, the increased LV wall thickness (by 34%), decreased LV cavity size (internal diameter; by 18%) and increased contractility (by 21%) in HEMI:CAR group compared to WT:WT confirmed hypertrophic hypercontractile cardiomyopathy in this symptomatic Fabry mouse model. At this age range, HR significantly increased (by 16%), stroke volume slightly decreased and there was no change in cardiac output.

### Non-symptomatic Fabry *Gla*KO mice exhibit mild cardiomegaly, mild cardiac dysfunction, with significant volume overload

**Fig 3:**
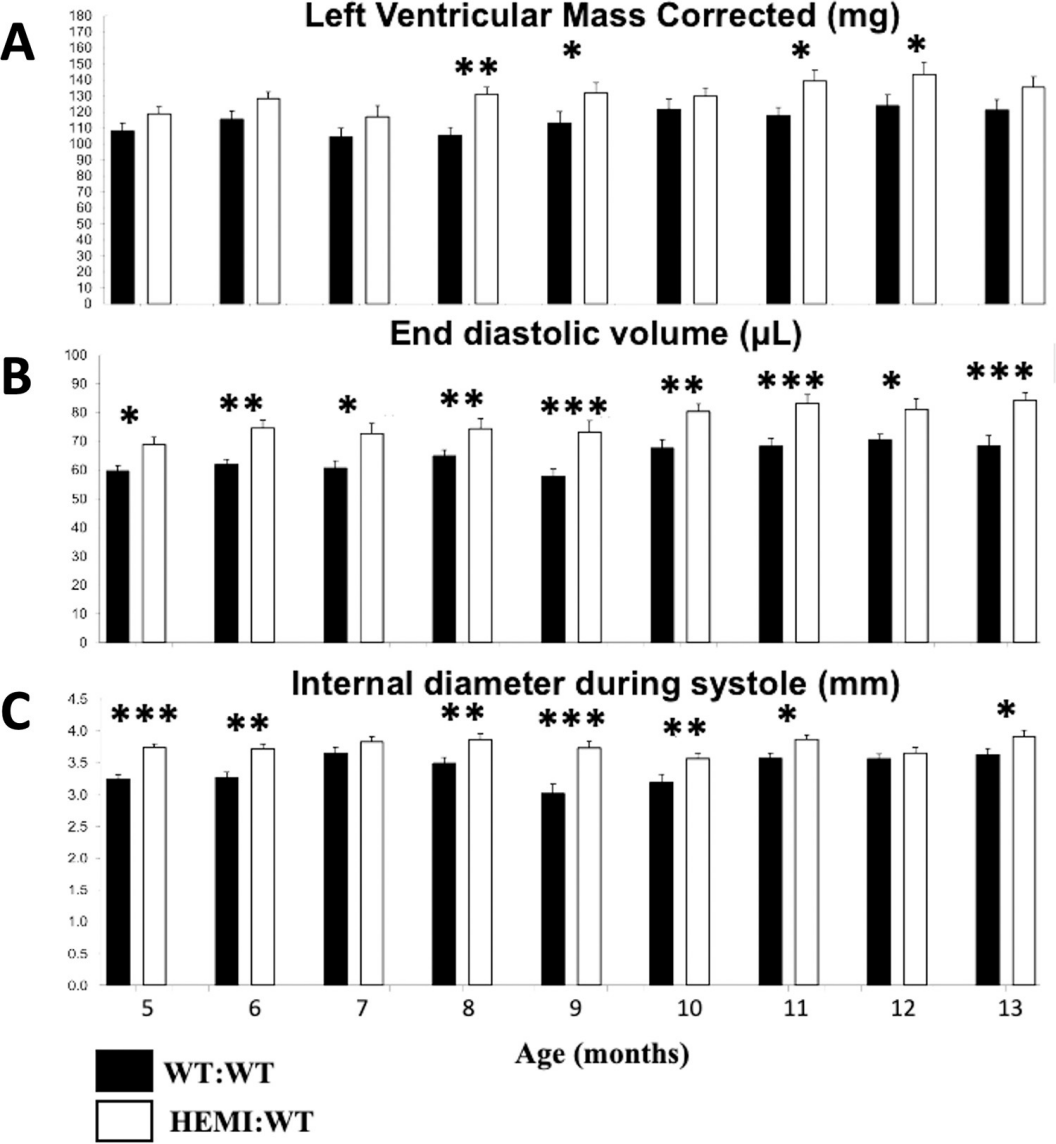
Longitudinal (5–13 months of age) assessments of LV mass, end-diastolic volume, and LV internal diameter indicate cardiomegaly and volume overload in *Gla*KO and age-matched WT mice. Number of animals per group = 20–21. Statistical analysis was performed using longitudinal mixed modeling and shown in S5 Table in S1 Appendix. * p ≤0.05, ** p ≤0.01, *** p ≤0.001. P values were adjusted using the Tukey method to control for Family Wise Error Rate (FWER).

**Fig 4:**
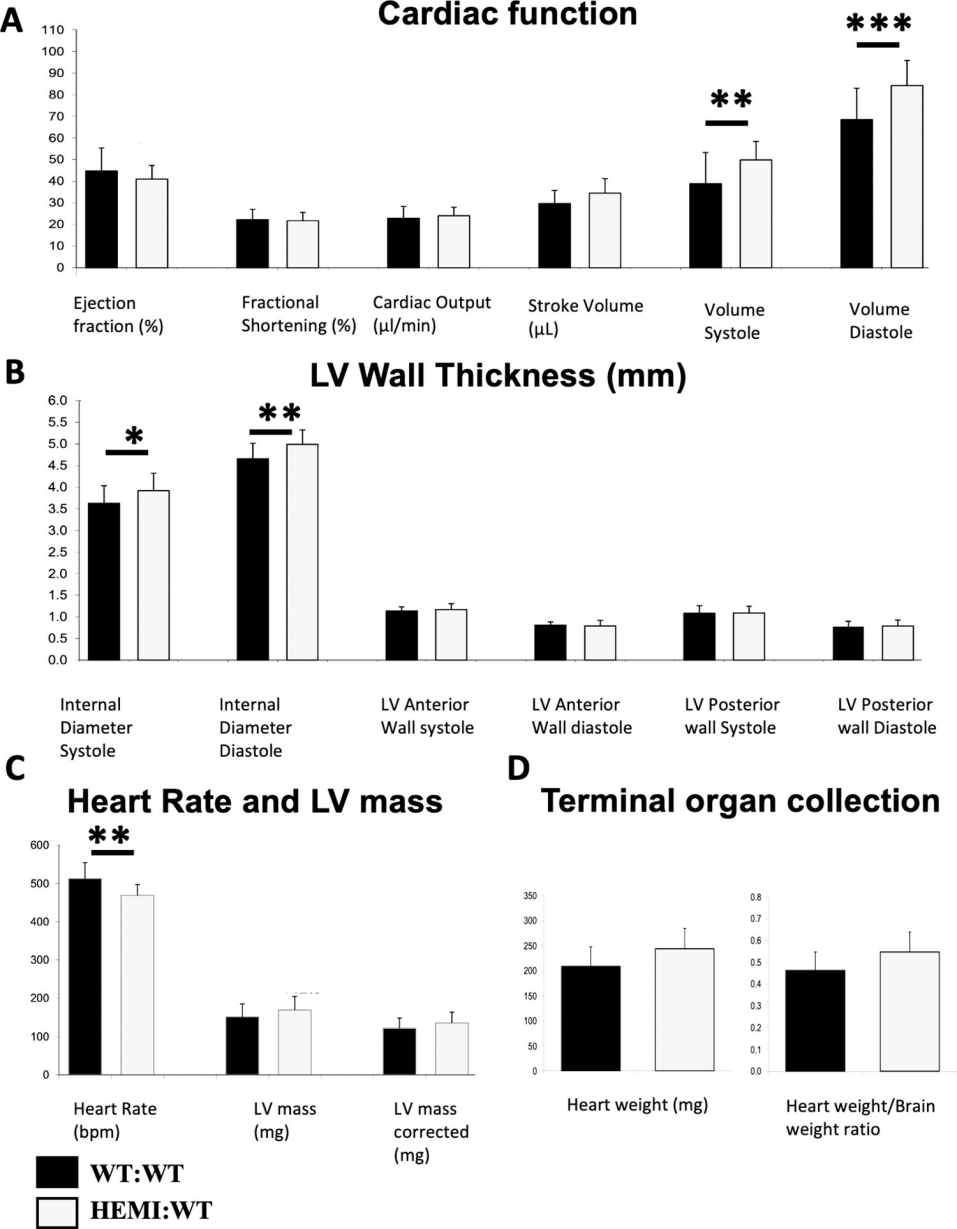
Characterization of cardiac function, LV wall thickness, heart rate, LV mass and organ weight in non-symptomatic *Gla*KO Fabry mice and age-matched WT controls at 13 months of age. Number of animals per group = 20–21. **A.** Cardiac function: Ejection fraction was lower in *Gla*KO mice (8% decrease) with no change in fractional shortening and cardiac output. Increase in stroke volume (16% increase), and significant increases in end-systolic volume (volume systole) and end-diastolic volume (volume diastole) by up to 28%, were observed in *Gla*KO mice. **B.** Cardiac wall thickness (LV anterior and posterior walls) of *Gla*KO mice were the same when compared to age-matched WT mice, but their LV internal diameter was significantly wider (up to 8% increase). **C.** LV mass of *Gla*KO mice was greater than WT (12% increase). Heart rate was significantly lower in *Gla*KO mice (9% decrease). **D.** Terminal organ collection. 13 months old mice show trend of increase in heart weight and in heart weight to brain weight Ratio (17% and 18% increase, respectively). Statistical analysis was performed using longitudinal mixed modeling across all ages, extracted for month 13, and shown in S6 Table in S1 Appendix. * p ≤0.05, ** p ≤0.01, *** p ≤0.001. Tukey method of p value correction was used to control for family wise error rate.

Longitudinal cardiac assessment of the HEMI:WT non-symptomatic Fabry *Gla*KO mice (Table 4, Study #2) revealed mild cardiomegaly, mild cardiac dysfunction, and yet a notable cardiac volume overload (Figs 3 and 4). Echocardiography showed that LV mass in *Gla*KO mice was higher than in the age-matched littermate WT:WT controls (ranging from 7% to 25% increase). Highest cardiomegaly was observed at 8 months of age (25% increase; P<0.01), although the difference did not achieve statistical significance at all time points (Fig 3A). Upon necropsy at 13 months, there was also a 17% increase in heart weight (HEMI:WT 243.9 ± 40.4 mg vs WT:WT 208.8 ± 39.1 mg; P = 0.09), with an 18% increase in the heart weight to brain weight ratio (P = 0.05; Fig 4D). From 5 to 13 months of age, *Gla*KO mice showed higher end-diastolic volume ranging from 15% to 27%, with the highest volume overload of 27% observed at 9 months of age (HEMI:WT 73.3 ± 17.1 μL vs WT:WT 57.8 ± 10.4 μL; P<0.001; Fig 3B).

At 13 months of age, *Gla*KO mice exhibited an 8% lower ejection fraction with no change in fractional shortening and cardiac output (Fig 4A). Notable was an increase in the stroke volume by 16% (P = 0.07), along with significant increases in end-systolic and end-diastolic volumes, by as much as 28%, when compared to the age-matched WT:WT controls (Fig 4A). The heart rate was found to be lower by 9% (P<0.05; Fig 4C). There was no change in cardiac wall thickness (LV anterior and posterior walls) at systole and diastole in these mice, whereas the LV internal diameter was significantly wider (expanding up to 24% increase at 9 months of age; P<0.001), when compared to age-matched controls (Figs 3C and 4B).

### Global longitudinal strain and radial strain

**Fig 5:**
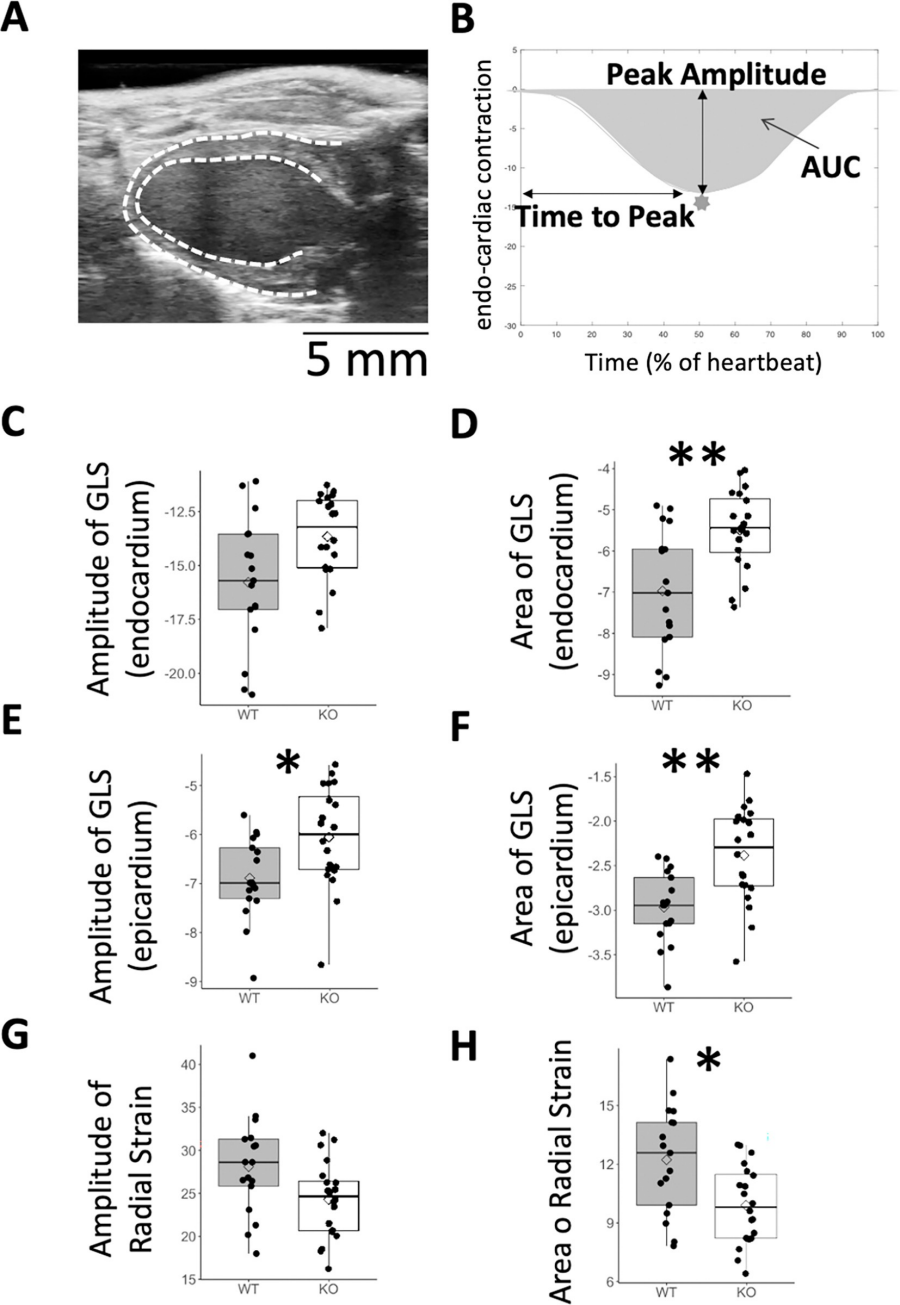
Compromised GLS and radial strain in *Gla*KO mice. **A.** Single frame from echocardiography scan (long axis), indicating endocardium and epicardium contours. **B.** example of a cardiac contraction over time during one heartbeat. **C, D.** peak amplitude and area under the curve (AUC) of global longitudinal strain (GLS) of endocardium. **E, F.** peak amplitude and AUC of GLS of epicardium. **G, H.** peak amplitude and AUC of radial strain. For stats, see S7 Table in S1 Appendix. * p≤0.05, ** p≤0.01, *** p≤0.001. p Values are Bonferroni corrected for multiple comparisons.

Endocardium and epicardium walls were manually segmented and automatically tracked from long axis echocardiography videos by means of Fujifilm Vevo-Strain software (Fig 5A). Fujifilm metadata were analyzed using Matlab and curves of GLS and Radial Strain were extracted. From each curve, we extracted Peak Amplitude, Area Under Curve (AUC) and Time to Peak (not shown), for HEM:WT and WT:WT mice at 13 months of age (example in Fig 5B). *Gla*KO mice exhibited lower (less negative) endocardial amplitude of the GLS (Fig 5C; P = 0.051), and area of GLS (Fig 5D; P<0.01), and lower epicardial amplitude of the GLS (Fig 5E; P<0.05), and area of GLS (Fig 5F; P<0.01). In addition, the Amplitude of the Radial Strain, and the area of the Radial Strain decreased in *Gla*KO mice when compared to WT controls (Fig 5G and 5H). Time to peak remained unchanged.

**Fig 6:**
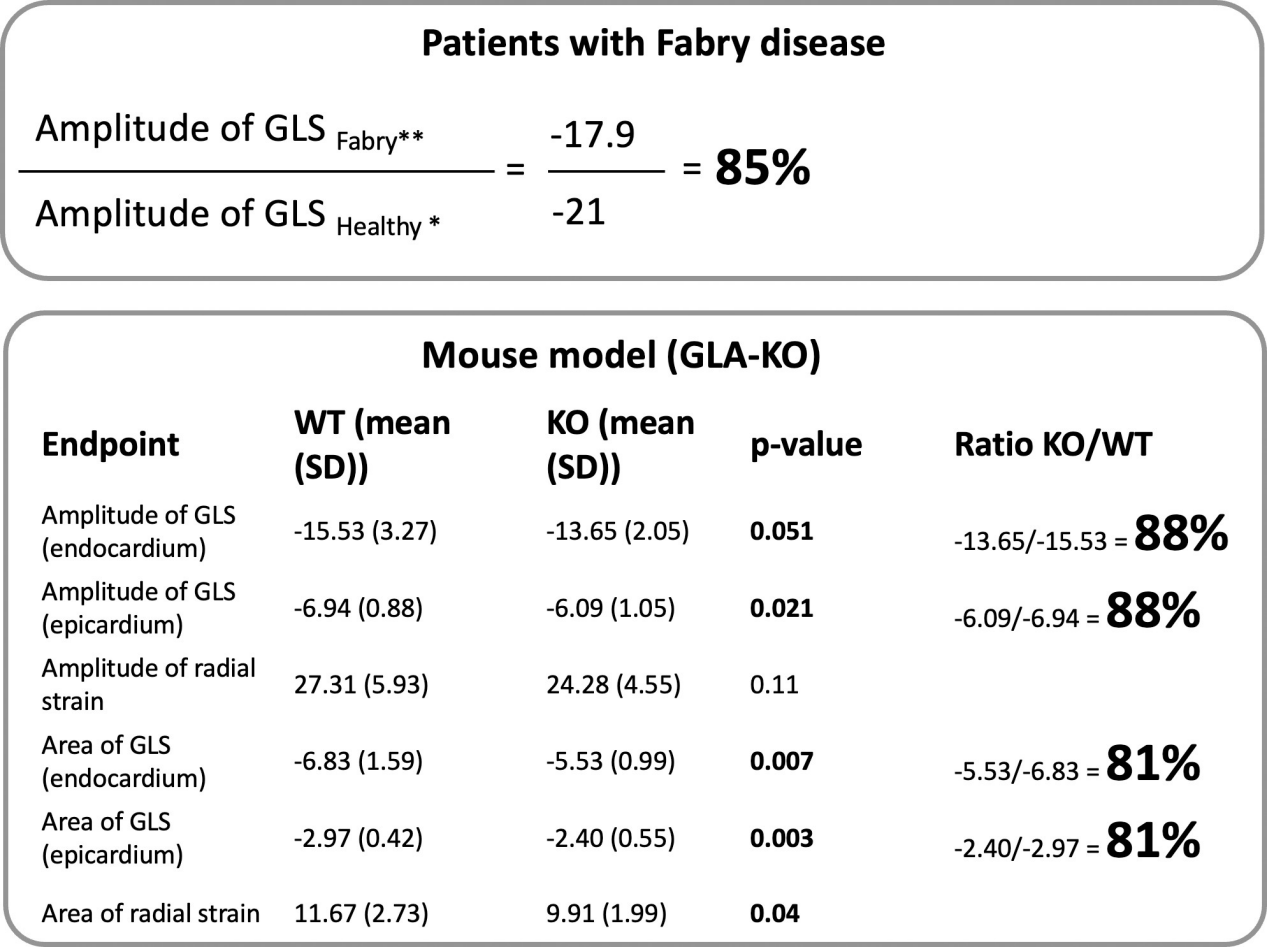
Translatability of strain measurements in mice.

The ratio between the GLS in *Gla*KO and in WT mice, spanning from 88% in case of the ratio of the amplitudes to 81% in case of the ratio of the areas (Fig 6) aligns with the Ratio (~85%) typically calculated by the literature, comparing cardiac strain values extracted from studies on FD [15, 16] with a meta-analysis of cardiac strain in the healthy population [17].

The deficit in GLS (amplitude and AUC in endocardium and epicardium) between *Gla*KO and WT:WT mice resembles the deficit in GLS measured in patients with FD when compared to the healthy population. The cardiac strain in *Gla*KO mice could be a good candidate for a translational biomarker.

## Discussion

In this study, we aimed to characterize the cardiac features of the available Fabry mouse models, namely alpha-galactosidase A-knockout *Gla*KO (HEMI:WT) and symptomatic G3Stg/*Gla*KO (HEMI:CAR). Our approach involved using echocardiography, heart weight and body weight measurements, and body scoring. The objective was to identify cardiac abnormalities in these mice, particularly those resembling early cardiac clinical changes observed in FD patients.

Our findings revealed that *Gla*KO mice exhibited several cardiac dysfunctions characteristic of the initial stages of Fabry cardiac pathology. Notably, cardiac structural and functional deficiencies could already be detected in 8 months old animals and for the most part persisted until the final study timepoint collected at 13 months of age. Echocardiography showed a significant increase in LV mass, peaking at a 25% increase by 8 months. At the terminal 13-month necropsy, we also observed an increase in heart weight (17%) and heart weight/brain weight ratio (18%). Volume overload reached its maximum at 9 months, with a 27% increase. Progressive widening of the LV internal diameter also peaked at 9 months with a 24% increase. Additionally, several other cardiac function parameters were affected to varying degrees.

Of particular interest, we noted a reduction in GLS, a myocardial deformation marker recently identified for early Fabry cardiomyopathy and used in the latest clinical trial for Fabry gene therapy [18, 19]. This GLS reduction in *Gla*KO mice mirrored what is observed in FD patients, suggesting its potential as a valuable translational biomarker candidate for assessing the cardiac efficacy of novel therapeutic approaches. However, the precise nature and implications of cardiac strain alterations, as well as their potential correlation with other disease manifestations of FD, remain areas for further exploration. The possibility of cardiac strain alterations being early indicators of FD makes this an exciting area for future research and may have significant implications for early disease detection and intervention strategies in human patients. This warrants further, in-depth investigation, potentially incorporating longitudinal studies and more extensive cardiac monitoring.

However, in the case of the G3Stg/*Gla*KO mice at 28 weeks of age, while they displayed hypertrophic cardiomyopathy characteristic of FD detected through increased LV wall thickness, several cardiac changes detected by echocardiography did not align with those seen in FD patients. For instance, both LV mass and heart weight were lower than in their WT littermate controls, contrary to findings seen in these patients. Taguchi et al. [13] also reported that this mouse line showed the manifestations which were not found in FD patients such as body weight loss and neurological abnormalities after 20 weeks of age. At younger age of 15–21 weeks, the increased LV wall thickness and decreased LV cavity size confirmed hypertrophic cardiomyopathy in this symptomatic Fabry mouse model. In addition, LV mass normalized to body weight was significantly higher in these mice by 20%, when compared to WT controls. These are the same features observed mainly in LV hypertrophy–positive FD patients with high LV mass index [19]. Our study limitations include the lack of determination of the cardiac manifestations of this mouse line at younger age of 5–14 weeks. The increase in LV wall thickness likely contributed to the observed increase in force of contraction (significant increase in ejection fraction and fractional shortening) in G3Stg/*Gla*KO mice, which is also not reported in patients. Furthermore, G3Stg/*Gla*KO mice exhibited high body scores and rapid health deterioration and significantly reduced lifespan, likely attributed to significant CNS Gb3 accumulation [13].

Histological assessment of the G3Stg/*Gla*KO mice and comparison to patient pathology has been presented by us at the WORLDSymposium 2023 [20], as well as in several papers published by others. In addition, evaluation of various histological biomarkers in the G3Stg/*Gla*KO mouse model, including collagen I was included in the recently submitted paper under review by our group [21]. We have demonstrated that immunostaining of cardiac sections revealed increase in Collagen I in G3Stg/*Gla*KO Fabry mice at 28 weeks of age. β-catenin is expressed in cardiomyocytes and the role of its aberrant signaling in fibrosis, arrhythmias and hypertrophy in different cardiac diseases has been well established. There were no reports on the contribution of β-catenin signaling to Fabry cardiac hypertrophy. We detected increase of β-catenin staining in the intercalated discs of G3Stg/*Gla*KO mice potentially indicating progression to hypertrophy. The accumulation of Gb3 was indirectly detected through increase of the cellular lysosomal compartment using LAMP1 lysosomal marker. The hematoxylin and eosin (H&E) staining of the G3Stg/*Gla*KO mice heart indicates morphological disarray [21].

Fabry cardiac phenotype development is different at different disease stages. Therefore, although, we believe that G3Stg/*Gla*KO could be a better model than *Gla*KO for testing cardiac Gb3 clearance [20], given these echocardiography findings, we conclude that G3Stg/*Gla*KO Fabry mice may be a less suitable model for translational studies assessing functional and structural cardiac efficacy of novel therapies. Our study highlights the importance of systematic longitudinal characterization of disease pathological manifestations in animal models to identify relevant nonclinical biomarkers and endpoints, while recognizing the limitations inherent to such models.

## Conclusion

Our research findings provide an in-depth characterization of FD progression in two genetically modified mouse models, spanning a wide range of biological domains, including body score phenotype, body and heart weights, cardiac features, and cardiac strain analysis. We found that only some cardiac findings in the G3Stg/*Gla*KO mice did align with the results reported in FD patients. Whereas *Gla*KO mice in the 8–13 month age range exhibited cardiac dysfunction indicative of their suitability as a translational model for evaluating the cardiac efficacy of novel treatments in reversing early cardiac pathology in FD patients. The discovery of cardiac strain alterations in *Gla*KO mice particularly highlights the prospect of early disease detection in FD, opening a new avenue for research. However, it is crucial to approach these findings with a measure of caution, bearing in mind the need for more extensive studies to fully ascertain their implications. The complexity of cardiac phenotype of FD necessitates a multi-pronged approach, integrating different markers and measures to offer a comprehensive understanding of the disease. This study forms a robust foundation for such further exploration, with the hope of accelerating our progress towards improved diagnostic and therapeutic strategies for FD.

## Supporting information

S1 Appendix Supplementary Figures and Tables. (DOCX)

S1 File (PPTX)

S1 Data (XLSX)
