# Supplementary material for: Cardiac manifestations of Fabry disease in G3Stg/GlaKO and GlaKO mouse models–Translation to Fabry disease patients
Source: PLoS One. 2024 May 31;19(5):e0304415. doi: 10.1371/journal.pone.0304415 (PMC11142664; doi:10.1371/journal.pone.0304415)
Supplement: S1 Appendix — (DOCX) [file pone.0304415.s001.docx]

**S1 Fig. Study #2 cardiac assessments schedule.**

**

**

**S2 Fig. Percent (%) change in body weight.**

Number of animals per group = 14–15. Data have been normalized to initial timepoint. Bars indicate standard deviation. Points indicate the mean at each age. Slopes are statistically different (ANOVA type III, p<0.0001, S2 Table). Age-to-Age p values across the absolute weights in the three models are calculated using 2-sample student t-test and applying Bonferroni correction and shown separately in S1 Table.

**

**

**S3 Fig. Body scores over time.**

Number of animals per group = 14–15; Bars indicate standard deviation. Points indicate the mean at each age. Age-to-Age p values are calculated through two-sample student’s t-tests and Bonferroni corrected and are shown separately in S3 Table (* p ≤0.05, ** p ≤0.01, *** p ≤0.001).





**S1 Table**. Body weights at each timepoint between WT:WT and Fabry disease groups (HEMI:WT and HEMI:CAR)

| **Weeks of Age** | **Groups** | **N** | **Mean ± SD (g)** | **Groups significantly different from WT:WT** |
| --- | --- | --- | --- | --- |
| 15 | WT:WT | 15 | 29.33 ± 3.33 | HEMI:WT p=0.0055 |
|  | HEMI:WT | 15 | 33.62 ± 3.80 |  |
|  | HEMI:CAR | 14 | 29.30 ± 3.73 |  |
| 16 | WT:WT | 15 | 29.95 ± 3.33 | HEMI:WT p=0.0152 |
|  | HEMI:WT | 15 | 33.49 ± 3.39 |  |
|  | HEMI:CAR | 14 | 28.82 ± 3.45 |  |
| 17 | WT:WT | 15 | 30.27 ± 3.52 | HEMI:WT p=0.0788 |
|  | HEMI:WT | 15 | 33.06 ± 3.59 |  |
|  | HEMI:CAR | 14 | 28.98 ± 3.60 |  |
| 18 | WT:WT | 15 | 30.32 ± 3.63 | HEMI:WT p=0.0422 |
|  | HEMI:WT | 15 | 33.57 ± 3.66 |  |
|  | HEMI:CAR | 14 | 28.93 ± 3.65 |  |
| 19 | WT:WT | 15 | 30.81 ± 3.66 | None |
|  | HEMI:WT | 15 | 33.45 ± 3.50 |  |
|  | HEMI:CAR | 14 | 28.31 ± 3.74 |  |
| 20 | WT:WT | 15 | 31.63 ± 3.62 | None |
|  | HEMI:WT | 15 | 33.84 ± 3.47 |  |
|  | HEMI:CAR | 13 | 28.39 ± 3.95 |  |
| 21 | WT:WT | 15 | 30.95 ± 3.68 | HEMI:WT p=0.0277 |
|  | HEMI:WT | 15 | 34.59 ± 3.92 |  |
|  | HEMI:CAR | 13 | 28.55 ± 4.17 |  |
| 22 | WT:WT | 15 | 32.01 ± 3.83 | HEMI:CAR p=0.0289 |
|  | HEMI:WT | 15 | 34.07 ± 4.06 |  |
|  | HEMI:CAR | 13 | 27.80 ± 4.69 |  |
| 23 | WT:WT | 15 | 32.51 ± 3.89 | HEMI:CAR p=0.0242 |
|  | HEMI:WT | 15 | 35.01 ± 4.51 |  |
|  | HEMI:CAR | 13 | 28.18 ± 4.63 |  |
| 24 | WT:WT | 15 | 32.95 ± 3.89 | HEMI:CAR p=0.0022 |
|  | HEMI:WT | 15 | 34.97 ± 4.70 |  |
|  | HEMI:CAR | 13 | 27.17 ± 4.44 |  |
| 25 | WT:WT | 15 | 32.72 ± 3.84 | HEMI:CAR p=0.0487 |
|  | HEMI:WT | 15 | 35.54 ± 4.85 |  |
|  | HEMI:CAR | 10 | 29.37 ± 2.61 |  |
| 26 | WT:WT | 15 | 33.69 ± 4.00 | HEMI:CAR p=0.0336 |
|  | HEMI:WT | 15 | 35.83 ± 5.15 |  |
|  | HEMI:CAR | 9 | 29.89 ± 3.01 |  |
| 27 | WT:WT | 15 | 33.21 ± 4.01 | None |
|  | HEMI:WT | 15 | 36.56 ± 5.44 |  |
|  | HEMI:CAR | 7 | 29.39 ± 2.69 |  |
| 28 | WT:WT | 15 | 33.73 ± 4.03 | HEMI:CAR p=0.0434 |
|  | HEMI:WT | 15 | 36.93 ± 5.18 |  |
|  | HEMI:CAR | 7 | 29.57 ± 2.58 |  |
| 29 | WT:WT | 15 | 34.60 ± 3.97 | HEMI:CAR p= 0.0127 |
|  | HEMI:WT | 15 | 37.57 ± 5.27 |  |
|  | HEMI:CAR | 7 | 29.56 ± 2.60 |  |
| 30 | WT:WT | 15 | 35.33 ± 4.12 | HEMI:CAR p=0.0015 |
|  | HEMI:WT | 15 | 37.61 ± 5.53 |  |
|  | HEMI:CAR | 7 | 28.57 ± 2.45 |  |

**S2 Table.** Longitudinal model of body weights over time between WT:WT and Fabry disease groups (HEMI:WT and HEMI:CAR)

| **Groups** | **Slope for each group independently** | **Overall group term** | | **Time term** | |
| --- | --- | --- | --- | --- | --- |
|  |  | **β** | **P-value** | **β** | **p-value** |
| WT:WT | 0.363 | -2.22 | 0.026 | 16.53 | P<0.0001 |
| HEMI:WT | 0.308 |  |  |  |  |
| HEMI:CAR | -0.171 |  |  |  |  |

**S3 Table.** Body scores at each timepoint between WT:WT and Fabry disease groups (HEMI:WT and HEMI:CAR). The p values are Bonferroni corrected

| **Weeks of Age** | **Groups** | **N** | **Mean ± SD** | **Groups significantly different from WT:WT** |
| --- | --- | --- | --- | --- |
| 16 | WT:WT | 15 | 6.00 ± 0.00 | HEMI:CAR p=0.024 |
|  | HEMI:WT | 15 | 6.07 ± 0.26 |  |
|  | HEMI:CAR | 14 | 6.64 ± 0.93 |  |
| 17 | WT:WT | 15 | 6.00 ± 0.00 | HEMI:CAR p=0.023 |
|  | HEMI:WT | 15 | 6.00 ± 0.00 |  |
|  | HEMI:CAR | 14 | 6.93 ± 1.33 |  |
| 18 | WT:WT | 15 | 6.00 ± 0.00 | HEMI:CAR p=0.00001 |
|  | HEMI:WT | 15 | 6.13 ± 0.35 |  |
|  | HEMI:CAR | 14 | 8.21 ± 1.53 |  |
| 19 | WT:WT | 15 | 6.00 ± 0.00 | HEMI:CAR P<0.0001 |
|  | HEMI:WT | 15 | 6.07 ± 0.26 |  |
|  | HEMI:CAR | 14 | 9.14 ± 1.83 |  |
| 20 | WT:WT | 15 | 6.00 ± 0.00 | HEMI:CAR p <0.0001 |
|  | HEMI:WT | 15 | 6.07 ± 0.26 |  |
|  | HEMI:CAR | 13 | 9.15 ± 1.63 |  |
| 21 | WT:WT | 15 | 6.00 ± 0.00 | HEMI:CAR p<0.0001 |
|  | HEMI:WT | 15 | 6.07 ± 0.26 |  |
|  | HEMI:CAR | 13 | 9.85 ± 2.51 |  |
| 22 | WT:WT | 15 | 6.00 ± 0.00 | HEMI:CAR p<0.0001 |
|  | HEMI:WT | 15 | 6.27 ± 0.46 |  |
|  | HEMI:CAR | 13 | 10.08 ± 2.47 |  |
| 23 | WT:WT | 15 | 6.07 ± 0.26 | HEMI:WT p=0.0002  HEMI:CAR p<0.0001 |
|  | HEMI:WT | 15 | 6.80 ± 0.56 |  |
|  | HEMI:CAR | 13 | 11.54 ± 2.67 |  |
| 24 | WT:WT | 15 | 6.00 ± 0.00 | HEMI:WT p=0.0007  HEMI:CAR P<0.0001 |
|  | HEMI:WT | 15 | 6.93 ± 0.88 |  |
|  | HEMI:CAR | 12 | 12.08 ± 2.84 |  |
| 25 | WT:WT | 15 | 6.00 ± 0.00 | HEMI:WT p=0.0002  HEMI:CAR p<0.0001 |
|  | HEMI:WT | 15 | 6.93 ± 0.80 |  |
|  | HEMI:CAR | 10 | 11.70 ±1.42 |  |
| 26 | WT:WT | 15 | 6.00 ± 0.00 | HEMI:WT p<0.0001  HEMI:CAR p<0.0001 |
|  | HEMI:WT | 15 | 7.47 ± 0.83 |  |
|  | HEMI:CAR | 9 | 12.56 ± 1.59 |  |
| 27 | WT:WT | 15 | 6.13 ± 0.35 | HEMI:WT p<0.0001  HEMI:CAR p<0.0001 |
|  | HEMI:WT | 15 | 7.47 ± 0.83 |  |
|  | HEMI:CAR | 9 | 13.44 ± 1.67 |  |
| 28 | WT:WT | 15 | 6.07 ± 0.26 | HEMI:WT p<0.0001  CAR p<0.0001 |
|  | HEMI:WT | 15 | 7.33 ± 0.72 |  |
|  | HEMI:CAR | 7 | 13.29 ± 0.76 |  |
| 29 | WT:WT | 15 | 6.00 ± 0.00 | HEMI:WT p<0.0001  HEMI:CAR p<0.0001 |
|  | HEMI:WT | 15 | 7.27 ± 0.59 |  |
|  | HEMI:CAR | 7 | 13.14 ± 0.90 |  |
| 30 | WT:WT | 15 | 6.00 ± 0.00 | HEMI:WT p<0.0001  HEMI:CAR p<0.0001 |
|  | HEMI:WT | 15 | 7.47 ± 0.64 |  |
|  | HEMI:CAR | 7 | 14.14 ± 0.69 |  |

**S4 Table.** 2-sample Student t-tests (Welch’s t-test in case of unequal variances) in Study #1 (HEMI:CAR vs WT:WT), at terminal point (27-28 weeks). The p values have been Bonferroni corrected

| **Feature** | **WT:WT** | **HEMI:CAR** | **% Change** | **p-value** |
| --- | --- | --- | --- | --- |
| LV Anterior Wall at Systole | 1.18 ± 0.15 | 1.37 ± 0.18 | +16.17% | 0.00016 |
| LV Anterior Wall at Diastole | 0.84 ± 0.11 | 0.93 ± 0.15 | +11.23% | 0.017 |
| LV Posterior wall at Systole | 1.04 ± 0.23 | 1.21 ± 0.26 | +15.70% | 0.022 |
| LV Posterior wall at Diastole | 0.80 ± 0.18 | 0.84 ± 0.24 | +5.94% | 0.43 |
| LV Internal Diameter at Systole | 3.50 ± 0.38 | 2.35 ± 0.50 | -32.87% | 7e-12 |
| LV Internal Diameter at Diastole | 4.45 ± 0.27 | 3.53 ± 0.48 | -20.72% | 3.15e-10 |
| LV Mass | 145.76 ± 32.76 | 112.42 ± 34.69 | -22.87% | 0.00104 |
| Corrected LV Mass | 116.61 ± 26.21 | 89.93 ± 27.75 | -22.87% | 0.00104 |
| Heart Weight | 174.80 ± 26.16 | 130.40 ± 30.89 | -25.40% | 1.63e-06 |
| Brain Weight | 454.04 ± 28.41 | 415.60 ± 31.76 | -8.47% | 4.26e-05 |
| Terminal Body Weight | 36.21 ± 4.65 | 22.79 ± 6.20 | -37.07% | 4.21e-11 |
| Ejection fraction | 42.02 ±11.40 | 58.82 ± 10.71 | +39.97% | 9.23e-10 |
| Fractional Shortening | 21.54 ± 5.74 | 34.09 ± 6.54 | +58.24% | 3.82e-09 |
| Stroke Volume | 28.94 ± 10.25 | 24.05 ± 5.90 | -16.92% | 0.0103 |
| Cardiac Output | 17.81 ± 4.21 | 15.66 ± 3.33 | -12.08% | 0.051 |
| Heart Rate | 461.46 ± 48.72 | 473.55 ± 67.60 | +2.62% | 0.47 |

**S5 Table.** Longitudinal analysis of data from Study #2 (HEMI:WT vs WT:WT). Data have been modeled using linear mixed modeling after log transformation, then deriving contrasts.

log(feature) ~ genotype * month + (1|mouse)

Genotype and month are both factors. The p values are adjusted using the Tukey method to control the family wise error rate

**LV Mass Corrected**

| **contrast** | **Month** | **ratio** | **SE** | **p value** | **lower CL** | **upper CL** | **sig** |
| --- | --- | --- | --- | --- | --- | --- | --- |
| Hemi:WT/WT:WT | 5 | 1.105 | 0.074 | 0.133 | 0.970 | 1.260 | Not Significant |
| Hemi:WT/WT:WT | 6 | 1.097 | 0.073 | 0.164 | 0.963 | 1.251 | Not Significant |
| Hemi:WT/WT:WT | 7 | 1.102 | 0.084 | 0.202 | 0.949 | 1.281 | Not Significant |
| Hemi:WT/WT:WT | 8 | 1.265 | 0.084 | 0.0001 | 1.110 | 1.441 | Significant |
| Hemi:WT/WT:WT | 9 | 1.154 | 0.078 | 0.035 | 1.010 | 1.317 | Significant |
| Hemi:WT/WT:WT | 10 | 1.050 | 0.067 | 0.441 | 0.927 | 1.191 | Not Significant |
| Hemi:WT/WT:WT | 11 | 1.174 | 0.075 | 0.013 | 1.035 | 1.331 | Significant |
| Hemi:WT/WT:WT | 12 | 1.149 | 0.071 | 0.026 | 1.017 | 1.298 | Significant |
| Hemi:WT/WT:WT | 13 | 1.101 | 0.071 | 0.138 | 0.970 | 1.249 | Not Significant |

**End Diastolic Volume**

| **contrast** | **Month** | **ratio** | **SE** | **p value** | **lower CL** | **upper CL** | **sig** |
| --- | --- | --- | --- | --- | --- | --- | --- |
| Hemi:WT/WT:WT | 5 | 1.150 | 0.065 | 0.014 | 1.029 | 1.285 | Significant |
| Hemi:WT/WT:WT | 6 | 1.192 | 0.068 | 0.002 | 1.065 | 1.334 | Significant |
| Hemi:WT/WT:WT | 7 | 1.179 | 0.072 | 0.008 | 1.044 | 1.330 | Significant |
| Hemi:WT/WT:WT | 8 | 1.164 | 0.063 | 0.006 | 1.046 | 1.295 | Significant |
| Hemi:WT/WT:WT | 9 | 1.257 | 0.073 | 0.0001 | 1.121 | 1.410 | Significant |
| Hemi:WT/WT:WT | 10 | 1.178 | 0.062 | 0.002 | 1.062 | 1.307 | Significant |
| Hemi:WT/WT:WT | 11 | 1.200 | 0.063 | 0.001 | 1.082 | 1.331 | Significant |
| Hemi:WT/WT:WT | 12 | 1.145 | 0.060 | 0.010 | 1.033 | 1.270 | Significant |
| Hemi:WT/WT:WT | 13 | 1.200 | 0.064 | 0.001 | 1.081 | 1.334 | Significant |

**LV Internal Diameter during Systole**

| **contrast** | **Month** | **ratio** | **SE** | **p value** | **lower CL** | **upper CL** | **sig** |
| --- | --- | --- | --- | --- | --- | --- | --- |
| Hemi:WT/WT:WT | 5 | 1.152 | 0.039 | 0.000 | 1.078 | 1.232 | Significant |
| Hemi:WT/WT:WT | 6 | 1.134 | 0.041 | 0.000 | 1.057 | 1.217 | Significant |
| Hemi:WT/WT:WT | 7 | 1.037 | 0.038 | 0.323 | 0.965 | 1.113 | Not Significant |
| Hemi:WT/WT:WT | 8 | 1.113 | 0.037 | 0.001 | 1.043 | 1.188 | Significant |
| Hemi:WT/WT:WT | 9 | 1.232 | 0.047 | 0.000 | 1.143 | 1.328 | Significant |
| Hemi:WT/WT:WT | 10 | 1.110 | 0.040 | 0.004 | 1.034 | 1.191 | Significant |
| Hemi:WT/WT:WT | 11 | 1.081 | 0.036 | 0.021 | 1.012 | 1.155 | Significant |
| Hemi:WT/WT:WT | 12 | 1.026 | 0.035 | 0.449 | 0.959 | 1.098 | Not Significant |
| Hemi:WT/WT:WT | 13 | 1.074 | 0.036 | 0.036 | 1.005 | 1.147 | Significant |

**S6 Table.** Analysis of terminal timepoint from Study #2 (HEMI:WT vs WT:WT). Data have been modeled from linear mixed modeling after log transformation (using all the dataset across months), then deriving contrasts for 13 months of age. The p values are adjusted using the Tukey method to control the family wise error rate

**Ejection Fraction (at 13 months of age)**

| **contrast** | **Month** | **Ratio** | **SE** | **p value** | **lower CL** | **upper CL** | **sig** |
| --- | --- | --- | --- | --- | --- | --- | --- |
| Hemi:WT/WT:WT | 13 | 0.908 | 0.057 | 0.124 | 0.802 | 1.027 | Not Significant |

**Fractional Shortening (at 13 months of age)**

| **contrast** | **Month** | **ratio** | **SE** | **p value** | **lower CL** | **upper CL** | **sig** |
| --- | --- | --- | --- | --- | --- | --- | --- |
| Hemi:WT/WT:WT | 13 | 0.977 | 0.068 | 0.736 | 0.851 | 1.121 | Not Significant |

**Cardiac Output (at 13 months of age)**

| **contrast** | **Month** | **ratio** | **SE** | **p value** | **lower CL** | **upper CL** | **sig** |
| --- | --- | --- | --- | --- | --- | --- | --- |
| Hemi:WT/WT:WT | 13 | 1.034 | 0.066 | 0.607 | 0.911 | 1.173 | Not Significant |

**Stroke Volume (at 13 months of age)**

| **contrast** | **Month** | **ratio** | **SE** | **p value** | **lower CL** | **upper CL** | **sig** |
| --- | --- | --- | --- | --- | --- | --- | --- |
| Hemi:WT/WT:WT | 13 | 1.132 | 0.082 | 0.087 | 0.982 | 1.305 | Not Significant |

**End-Systolic Volume (at 13 months of age)**

| **contrast** | **Month** | **ratio** | **SE** | **p value** | **lower CL** | **upper CL** | **sig** |
| --- | --- | --- | --- | --- | --- | --- | --- |
| Hemi:WT/WT:WT | 13 | 1.247 | 0.095 | 0.004 | 1.073 | 1.448 | Significant |

**End-Diastolic Volume (at 13 months of age)**

| **contrast** | **Month** | **ratio** | **SE** | **p value** | **lower CL** | **upper CL** | **sig** |
| --- | --- | --- | --- | --- | --- | --- | --- |
| Hemi:WT/WT:WT | 13 | 1.200 | 0.064 | 0.001 | 1.081 | 1.334 | Significant |

**Internal Diameter Systole (at 13 months of age)**

| **contrast** | **Month** | **ratio** | **SE** | **p value** | **lower CL** | **upper CL** | **sig** |
| --- | --- | --- | --- | --- | --- | --- | --- |
| Hemi:WT/WT:WT | 13 | 1.074 | 0.036 | 0.036 | 1.005 | 1.147 | Significant |

**Internal Diameter Diastole** **(at 13 months of age)**

| **contrast** | **Month** | **ratio** | **SE** | **p value** | **lower CL** | **upper CL** | **sig** |
| --- | --- | --- | --- | --- | --- | --- | --- |
| Hemi:WT/WT:WT | 13 | 1.066 | 0.024 | 0.006 | 1.019 | 1.115 | Significant |

**LV Anterior Wall Systole (at 13 months of age)**

| **contrast** | **Month** | **ratio** | **SE** | **p value** | **lower CL** | **upper CL** | **sig** |
| --- | --- | --- | --- | --- | --- | --- | --- |
| Hemi:WT/WT:WT | 13 | 1.029 | 0.045 | 0.510 | 0.945 | 1.120 | Not Significant |

**LV Anterior Wall Diastole (at 13 months of age)**

| **contrast** | **Month** | **ratio** | **SE** | **p value** | **lower CL** | **upper CL** | **sig** |
| --- | --- | --- | --- | --- | --- | --- | --- |
| Hemi:WT/WT:WT | 13 | 0.985 | 0.051 | 0.762 | 0.890 | 1.089 | Not Significant |

**LV Posterior Wall Systole (at 13 months of age)**

| **contrast** | **Month** | **ratio** | **SE** | **p value** | **lower CL** | **upper CL** | **sig** |
| --- | --- | --- | --- | --- | --- | --- | --- |
| Hemi:WT/WT:WT | 13 | 1.005 | 0.057 | 0.924 | 0.899 | 1.124 | Not Significant |

**LV Posterior Wall Diastole (at 13 months of age)**

| **contrast** | **Month** | **ratio** | **SE** | **p value** | **lower CL** | **upper CL** | **sig** |
| --- | --- | --- | --- | --- | --- | --- | --- |
| Hemi:WT/WT:WT | 13 | 1.012 | 0.071 | 0.869 | 0.881 | 1.161 | Not Significant |

**Heart Rate (at 13 months of age)**

| **contrast** | **Month** | **ratio** | **SE** | **p value** | **lower CL** | **upper CL** | **sig** |
| --- | --- | --- | --- | --- | --- | --- | --- |
| Hemi:WT/WT:WT | 13 | 0.918 | 0.029 | 0.008 | 0.862 | 0.978 | Significant |

**LV Mass (at 13 months of age)**

| **contrast** | **Month** | **ratio** | **SE** | **p value** | **lower CL** | **upper CL** | **sig** |
| --- | --- | --- | --- | --- | --- | --- | --- |
| Hemi:WT/WT:WT | 13 | 1.101 | 0.071 | 0.138 | 0.970 | 1.249 | Not Significant |

**LV Mass Corrected (at 13 months of age)**

| **contrast** | **Month** | **ratio** | **SE** | **p value** | **lower CL** | **upper CL** | **sig** |
| --- | --- | --- | --- | --- | --- | --- | --- |
| Hemi:WT/WT:WT | 13 | 1.101 | 0.071 | 0.138 | 0.970 | 1.249 | Not Significant |

**Heart Weight**

| **contrast** | **ratio** | **SE** | **t ratio** | **p value** | **lower CL** | **upper CL** | **sig** |
| --- | --- | --- | --- | --- | --- | --- | --- |
| Hemi:WT/WT:WT | 1.125 | 0.076 | 1.728 | 0.093 | 0.98 | 1.291 | Not Significant |

**Heart Weight / Brain Weight ratio**

| **contrast** | **ratio** | **SE** | **t ratio** | **p value** | **lower CL** | **upper CL** | **sig** |
| --- | --- | --- | --- | --- | --- | --- | --- |
| Hemi:WT/WT:WT | 1.139 | 0.074 | 1.995 | 0.054 | 0.998 | 1.3 | Not Significant |

**S7 Table.** P values have been calculated using 2-sample Student t-test (Welch’s t-test for unequal variances), and Bonferroni corrected

| **Feature** | **p-value** |
| --- | --- |
| Amplitude of GLS endocardium | 0.051 |
| Area of GLS endocardium | 0.007 |
| Amplitude of GLS epicardium | 0.021 |
| Area of GLS epicardium | 0.003 |
| Amplitude of radial strain | 0.11 |
| Area of radial strain | 0.04 |
