# Supplementary material for: Cardiac manifestations of Fabry disease in G3Stg/GlaKO and GlaKO mouse models–Translation to Fabry disease patients
Source: PLoS One. 2024 May 31;19(5):e0304415. doi: 10.1371/journal.pone.0304415 (PMC11142664; doi:10.1371/journal.pone.0304415)
Supplement: S1 File — (PPTX) [file pone.0304415.s002.pptx]

## Slide 1
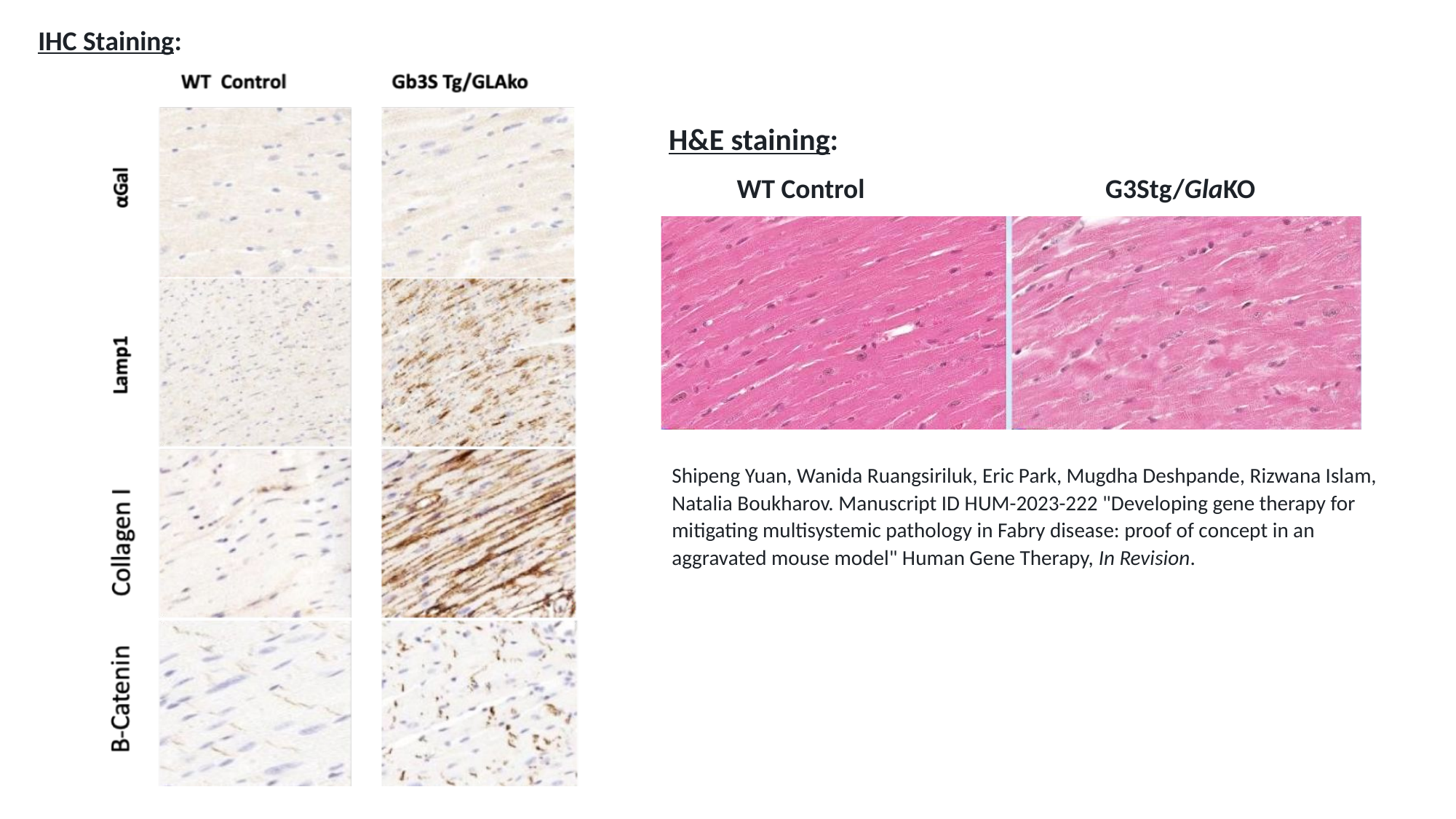

IHC Staining:
H&E staining:
 WT Control			G3Stg/GlaKO
Shipeng Yuan, Wanida Ruangsiriluk, Eric Park, Mugdha Deshpande, Rizwana Islam, Natalia Boukharov. Manuscript ID HUM-2023-222 "Developing gene therapy for mitigating multisystemic pathology in Fabry disease: proof of concept in an aggravated mouse model" Human Gene Therapy, In Revision.
